# Developing and testing tele-support psychotherapy using mobile phones for depression among youth in Kampala district, Uganda: study protocol for a pilot randomized controlled trial

**DOI:** 10.3389/fdgth.2025.1515193

**Published:** 2025-02-18

**Authors:** Etheldreda Nakimuli-Mpungu, Jeremiah Mutinye Kwesiga, John Mark Bwanika, Davis Musinguzi, Caroline Nakanyike, Jane Iya, Sabrina Bakeera Kitaka, Benedict Akimana, Charlotte Hawkins, Patricia Cavazos, Jean B. Nachega, Edward J. Mills, Musisi Seggane

**Affiliations:** ^1^ Department of Psychiatry, College of Health Sciences, Makerere University, Kampala, Uganda; ^2^ SEEK Group Support Psychotherapy Initiative, Kampala, Uganda; ^3^ The Medical Concierge Group, Kampala, Uganda; ^4^ Department of Pediatrics, College of Health Sciences, Makerere University, Kampala, Uganda; ^5^ Butabika National Referral Mental Hospital, Ministry of Health of Uganda, Kampala, Uganda; ^6^ University College London, London, United Kingdom; ^7^ Health and Behavior Research Center, School of Medicine in St Louis, Washington University, St. Louis, MO, United States; ^8^ Departments of Epidemiology, University of Pittsburgh Graduate School of Public Health, Pittsburgh, PA, United States; ^9^ Center for Infectious Disease, Department of Medicine, Stellenbosch University Faculty of Medicine and Health Sciences, Cape Town, South Africa; ^10^ Departments of International Health and Epidemiology, Bloomberg’s School of Public Health, Johns Hopkins University, Baltimore, MD, United States; ^11^ Department of Clinical Epidemiology & Biostatistics, McMaster University, Hamilton, ON, Canada

**Keywords:** tele-support psychotherapy, depression, COVID-19, youth, Uganda tele-support psychotherapy, randomized controlled trial, Uganda

## Abstract

**Introduction:**

In the post-COVID-19 era, depressive disorders among youth have risen significantly, creating an urgent need for accessible, cost-effective mental health interventions. This study adapts Group Support Psychotherapy into Tele-Support Psychotherapy (TSP) via mobile phones. It aims to evaluate its feasibility, acceptability, effectiveness, and cost-efficiency in addressing mild to moderate depression among youth in central Uganda.

**Methods and analysis:**

This study will use a mixed-methods approach, starting with a qualitative phase to adapt Group Support Psychotherapy into Tele-Support Psychotherapy (TSP) via mobile phones. Guided by ecological theories and the Unified Theory of Acceptance and Use of Technology (UTAUT), focus group discussions and interviews with youth, mental health professionals, and stakeholders will inform the development of a youth-tailored call platform integrated into Rocket Health Africa's telehealth services. Data will be analyzed using grounded theory and MAXQDA Analytics Pro 2022 to guide intervention adaptation. An open-label randomized controlled trial will enroll 300 youth (15–30 years) with mild to moderate depression from Kampala, Uganda, to evaluate Tele-Support Psychotherapy (TSP). Participants will be randomized to TSP with standard mental health services (SMHS) or SMHS alone. Primary outcomes include feasibility and acceptability, with secondary outcomes assessing cost-effectiveness, depressive symptom changes, and social support. Intention-to-treat analysis using structural equation modeling will evaluate treatment effects, complemented by qualitative insights into implementation barriers and facilitators.

**Discussion:**

This study protocol develops and evaluates Tele-Support Psychotherapy (TSP) for youth depression in resource-limited settings, addressing mental health gaps exacerbated by COVID-19. Using user-centered design and mixed methods, it explores TSP's feasibility, adaptability, and cost-effectiveness while addressing barriers like technology literacy, laying the groundwork for accessible digital mental health solutions.

**Trial Registration:**

PACTR202201684613316.

## Introduction

There is growing recognition of tele-psychotherapy as a viable and effective treatment for depression among youth, offering a convenient and accessible option for mental health care (1). This approach is crucial in addressing the increasing mental health issues, such as depression and associated stigma (2). Notably, many mental health problems are mild to moderate in severity and respond well to first-line psychological treatments (3).

Depression among youth in Kampala, particularly in vulnerable populations, is a critical concern exacerbated by socio-economic challenges, violence, and inadequate healthcare access, with young women disproportionately affected. A systematic review and meta-analysis found that the pooled prevalence of depression in Uganda is approximately 30.2%, with refugees experiencing the highest rates at 67.63% (4, 5). Experiences of sexual and physical violence, along with the SAVA syndemic (substance use, violence, and HIV/AIDS), further increase vulnerability to depression (6, 7). The lack of adequate healthcare access is a significant barrier to addressing mental health issues among these groups.

The Covid-19 pandemic has highlighted the need for telemedicine in low- and middle-income countries (LMICs), which face unique challenges due to strained healthcare systems and limited resources. Despite technological, regulatory, and economic challenges, there has been a high level of acceptance for telemedicine services in these regions during the pandemic (8) While online therapeutic services have been around long before the dawn of the COVID-19 pandemic in high-income countries (HICs), they were limited in low- and middle-income countries (9). Most mental health professionals in LMICs had to transition towards tele- psychotherapy for the first time to provide ongoing care (10). This new treatment modality makes it necessary for researchers in LMICs to start investigating and documenting the processes involved in delivering tele-psychotherapy and the treatment outcomes.

Digital mental health interventions for youth are emerging in sub-Saharan Africa. For instance, interventions like “Shamiri Digital” and “Kuamsha” have shown promise in reducing depressive symptoms, with “Shamiri Digital” demonstrating significant improvements through growth mindset, gratitude, and value affirmation techniques (11), and “Kuamsha” using a gamified, user-centred design for behavioural activation therapy (12). However, limited research in low-resource settings and the lack of standardized, culturally sensitive approaches emphasise the need for further rigorous evaluations and adaptations (13). A scoping review of 64 studies describing 57 mental health interventions for adolescents in sub-Saharan Africa revealed a significant gap in the use of digital mental health interventions (14). This underutilization highlights an opportunity for further research and development to assess the feasibility and effectiveness of incorporating digital technologies. Leveraging these tools could enhance mental health support for some adolescents in the region.

Digital health interventions (DHIs) in sub-Saharan Africa have shown promise in improving health outcomes across various domains, though effectiveness varies due to systemic challenges like lack of coordination, scalability, and infrastructure deficits (15, 16). For HIV care, telemedicine has improved palliative care communication, while ART adherence interventions show mixed results, with some success using SMS reminders (17, 18). mHealth interventions have facilitated care for NCDs, though their success depends on patient attitudes and resources (19). Similarly, mobile interventions have improved maternal health services in underserved areas but require better evaluation (20).

Tele-psychotherapy has proven to be an effective and accessible alternative to traditional in-person therapy for managing anxiety and depression, utilizing modalities such as telephone, video, and online platforms. Studies highlight the benefits of telephone-administered psychotherapy in reducing depression severity with high adherence rates (21) and mobile phone support in improving depression management outcomes (22). Computerized cognitive behavioral therapy (cCBT) has also shown effectiveness in reducing anxiety and depression symptoms, particularly with supervision to improve adherence and reduce dropout rates (23, 24). Young people in developing countries prefer interactive, game-like digital interventions with personalized and social features (25, 26), though low engagement and technical issues remain challenges. Co-designing these platforms with young people and caregivers enhances their acceptability and effectiveness, ensuring they meet user needs and preferences (27, 28).

Previously, we developed culturally appropriate group support psychotherapy to treat mild to moderate depression (29). Our comprehensive research demonstrated that these approaches effectively address depression and enhance physical health and can be administered by lay health workers, alleviating the critical shortage of mental health professionals available to provide services to those in need (30, 31). In this context, we collaborated with the Medical Concierge Group (TMCG) to transform group support psychotherapy (GSP) into tele-support psychotherapy (TSP), which is offered individually through mobile phones. TMCG, a prominent digital health enterprise in Uganda, has demonstrated the practicality, acceptance, and effects of tele-health in diverse medical fields. However, their offerings currently lack tele-psychotherapy (32). The specifics of setting up tele-psychotherapy and the results from such services remain largely unexplored.

The Unified Theory of Acceptance and Use of Technology (UTAUT) is a widely used framework for understanding the factors influencing technology adoption, focusing on performance expectancy, effort expectancy, social influence, and facilitating conditions as key determinants (33). Extensions of the model incorporate factors like trust, perceived enjoyment, and satisfaction, highlighting the need for context-specific adaptations to address variations in demographic and educational contexts (34, 35). Performance expectancy and ease of use consistently emerge as critical predictors of behavioural intention, while social influence and facilitating conditions play variable roles depending on the context (36, 37). The UTAUT model will be used to guide the development and evaluation of tele-support psychotherapy, as it identifies the factors influencing user acceptance of digital mental health interventions. By addressing these determinants, such as ensuring ease of use, cultural relevance, and accessible resources, the model will enhance the design and scalability of tele-support psychotherapy, ensuring it meets the needs and preferences of target populations, particularly youth.

Further, the Ecological Validity and Culturally Sensitive Framework is crucial in designing tele-psychotherapy interventions, especially in diverse cultural settings. This framework will ensure that TSP is tailored to the cultural and environmental contexts of the target population. This approach will not only respect the cultural nuances of the population but also increase the likelihood of successful implementation and acceptance of the intervention.

This paper outlines the protocol for development and evaluation of Tele-Support Psychotherapy (TSP, administered by lay counselors) using mobile phones, for treating mild to moderate depression among Ugandan youth. In the initial phase, we will conduct community-based participatory qualitative research to gather insights for developing a call platform and adapting Group Support Psychotherapy (GSP) into a Tele-Support Psychotherapy (TSP) model tailored for youth depression treatment. In the trial phase, the primary objective will be to evaluate the feasibility, and acceptability of TSP delivered via mobile phones by lay counsellors as a treatment for mild to moderate depression among youth in the Kampala district.

Further, we will determine whether TSP using mobile phones can be delivered by lay counselors as planned and also explore causal and contextual factors (barriers and facilitators) influencing access to TSP. Secondary objectives will include an exploration of the preliminary effectiveness and cost-effectiveness of tele-support psychotherapy using mobile phones in treating mild to moderate depression among youth. Further, we will explore mediators and moderators of the effect of TSP on depression. We hypothesize that compared to the control intervention, TSP using mobile phones will demonstrate better feasibility, acceptability, preliminary effectiveness, and cost-effectiveness. The primary endpoint will be six months from baseline. The data obtained will be used to design a definitive trial which will test the hypothesis that TSP added to SMHS leads to more reduction in depression symptoms than the sole use of SMHS among youth in Kampala district.

## Methods

### Study setting

The study will take place in three distinct areas within the Kampala district: the slums of Naguru Go-down and Kamwokya, and the student community at Makerere University. Kampala has 62 slums, accommodating half of the city's population on just 16% of its land. Kamwokya stands out as one of the most notorious slums, known for its high density, housing around 6,380 people, and grappling with inadequate infrastructure and significant public health issues. Naguru Go-down, located in central Kampala, comprises roughly 2,080 households, totalling an estimated population of 10,400, with an average household size of five individuals. Makerere University, Uganda's largest public university, hosts around 35,000 students aged 18–30. These communities, drawing youth from across the nation, are characterized by their diversity and high density, making them particularly susceptible to a range of public health challenges, including poverty, underemployment, financial and academic pressures, drug use, and overcrowding (38).

### Study design

During the preparatory phase, a qualitative study will be carried out. Co-principal investigator JMB, leveraging over ten years of connections with officials and health centers in the Kampala district, will facilitate introductions between the research team and important local figures. We plan to hold nine focus group discussions (FGDs) with youth, community health workers, and political representatives from the three target areas: Naguru, Kamwokya, and Makerere, each hosting 3 discussions. Additionally, key informant interviews (KIs) with a diverse group of 10 stakeholders, including psychologists, health officials, community and religious leaders, will explore their understanding, perspectives, and attitudes towards employing tele-psychotherapy for youth mental health issues. For the qualitative interviews, we will employ purposive sampling, conducting them in the local dialect. These interviews will be audio-recorded, then transcribed and, where necessary, translated word-for-word. Prior to the KIs and FGDs, informed consent will be secured from all participants.

In conjunction with these efforts, we will establish a Community Advisory Board (CAB) that includes diverse community representatives such as a community and youth leader, a faith healer, local council and student leaders, a community health worker, and a representative from key populations. The CAB's role is to ensure ongoing trust in the research activities within the community. CAB members will also be involved in the KIs, enhancing the research's credibility and providing essential feedback on the TSP model and study protocol, especially regarding mental health and psychosocial issues. Insights from these interviews and discussions will inform adjustments to the GSP content, making it suitable for TSP delivery to youth through mobile technology.

Collaboration will be sought with professional counsellors (*N* = 10) who will receive training in the delivery of TSP. Training workshops will also be used to review our theoretical framework and select psychotherapeutic skills from the GSP model to be used in the TSP model for youth. Further, we will refine lay health workers’ training materials and guides. These professionals will train, mentor and supervise 40 lay counsellors who will deliver tele-psychotherapy through a virtual platform to youth with mild to moderate depression.

In the trial phase, we shall conduct a pilot single-blinded randomized controlled trial to test the feasibility and

acceptability of using TSP in combination with standard mental health services(SMHS) vs. SMHS only to treat mild to moderate depression among youth in Kampala district. We aim to randomize 300 youth (1:1) to either both TSP and SMHS (*N* = 150) or SMHS only (*N* = 150). The 150 subjects in each arm will be recruited from three communities (Makerere University (*N* = 50), Kamwokya slums (*N* = 50) and Naguru go-down slums (*N* = 50) in each treatment arm and invited to participate. Participants will be evaluated at baseline, 6 and 12 months after baseline assessment.

A longitudinal process evaluation of the delivery of TSP using mobile phones and SMHS using mixed methods will run alongside the trial to assess acceptability, feasibility, fidelity, barriers and facilitators and how intervention recipients respond to the different intervention components. Open-ended exit interviews with recruited trained lay counsellors and study participants will seek to understand stressors in their communities, their own psychosocial support needs, and their knowledge regarding mental health after training, satisfaction, barriers and facilitators of participation in TSP sessions. Attendance registers and training logs will be used to track indicators of feasibility.

**Figure 1:**
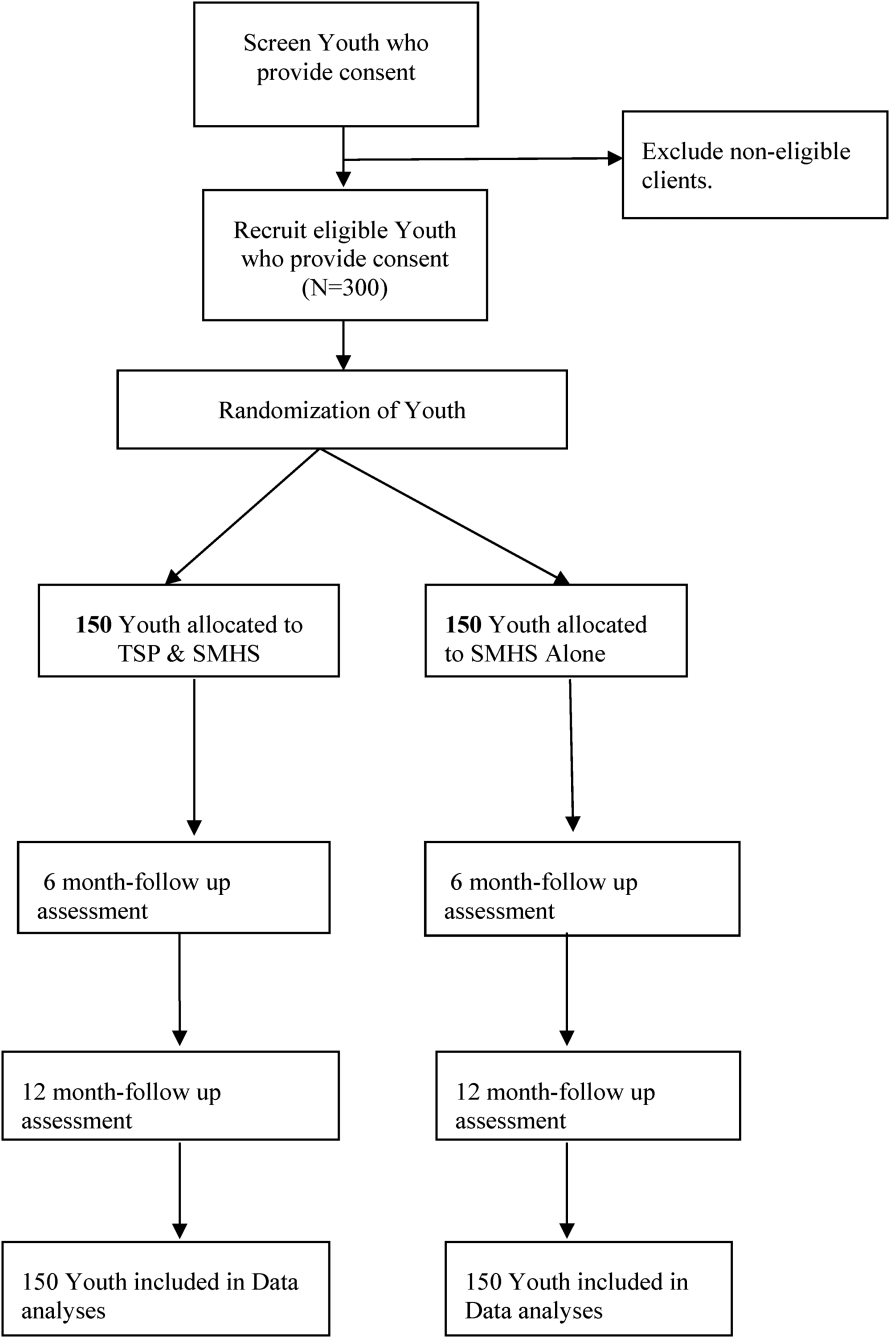
Participant flow diagram.

The study protocol is registered in the Pan African Clinical Trials Registry. The reporting of the trial will be in accordance with the Standard Protocol Items: Recommendations for Interventional Trials (SPIRIT) guidelines (39) for intervention trials (see Supplementary File S1) and the CONSORT statements for pilot randomized trials (40). The study was submitted to and approved by both the Makerere University College of Health Sciences Research Ethics Committee and the Uganda National Council of Science and Technology. All study participants will be required to provide written informed consent. Participants will receive a financial incentive to compensate for their time and defray internet data costs (if any). Figure 1 summarizes the trial profile.

### Participant eligibility criteria

To be eligible for the study, an individual must be aged 15–30 years, with a diagnosis of mild to moderate depression, reside in Kamwokya, Naguru and Makerere areas in Kampala district, have a mobile phone, provide written informed consent and be able to speak Luganda or English.

Individuals aged 15–17 years must be either a mature minor or an emancipated minor (mature and emancipated minors are permitted to independently provide informed consent to participate in research).

Individuals who were mentally and or physically disabled as evidenced by a score inferior to 50% on the Karnofsky performance scale will be excluded from the study.

### Recruitment

Equal numbers of young people (15–30 years) living in the Naguru Go-down and Kamwokya slums in Kampala and students from Makerere University will be recruited and assessed for eligibility. A member of our team will be responsible for linking all excluded individuals to mental health services. Research assistants (RAs) will work with TMCG staff to launch an online and offline mental health awareness campaign to create awareness about mental health problems and available treatment options, including standard mental health services provided at government health facilities and the newly developed tele-psychotherapy service.

In the online mental health awareness campaign, given the high ownership of mobile phones in this demographic, more publicity will be done through SMS messaging and social media. The youthful population of university students will be reached through TMCG's social media channels, which are popular amongst young people, including University students, for example, the Facebook page has 58,000 followers.

This project will be explained in layman's language, and young people interested in participating will be invited to contact the study team.

In the offline mental health awareness campaign, the study team will work in collaboration with local leaders in Kamwokya and Naguru Go-down, as well as health workers at the Health facilities providing mental health services to conduct community sensitization meetings in which they will raise awareness about mental health problems and available treatment options including standard mental health services provided at government health facilities and the newly developed tele-psychotherapy service. Also, the project objectives will be explained in layman's language. About six to eight community meetings will be held, and at the end of each meeting, young people interested in participating in the project will be invited to contact the study team. This will ensure that all study participants are exposed to communication campaigns and then can be invited to participate.

### Randomization and masking

A biostatistician will randomly assign participants to either an intervention or control group using specialized statistical software, ensuring impartiality as the biostatistician has no direct contact with participants. Randomized blocks of varying sizes will be used to enhance the balance between study arms and reducing sequence predictability. Participants, each assigned a unique study code for anonymity, will be identified only by their phone number and study code to the research team. This randomization will be applied across three distinct communities—Naguru, Kamyokya, and Makerere University—with 100 participants from each being randomized, ensuring the study's findings can be generalized across different population subsets. Although participants will know if they are receiving the experimental or control intervention, the nature of these interventions will be masked from independent outcome assessors and data analysts to prevent assessment bias and ensure objective outcome evaluation.

### Study interventions

#### Tele-support psychotherapy model

The structure of the TSP model will be designed to align closely with the GSP model. Detailed content will be determined from the qualitative research we plan to undertake in the preparatory phase of the trial. Generally, the initial session will focus on establishing a safe therapeutic environment, clarifying expectations and setting ground rules. The second session will aim to increase the client's awareness of common mental health issues like depression, their symptoms, causes, and treatments, and the importance of addressing them to prevent complications. Sessions three and four will allow the client to express and share personal distressing experiences, provide emotional support, and equip them with skills to manage negative thoughts. The fifth session will be centred on teaching and practicing positive coping skills to manage difficult situations and excessive worries while avoiding negative coping mechanisms. In the sixth session, the focus will shift to coping with stigma and discrimination at personal, family, and community levels. The seventh session aims to empower clients with basic livelihood skills to identify income-generating activities, enhancing their ability to control their lives post-therapy. The final session involves the presentation of these income-generating activities by the clients, where they receive feedback and are guided in creating a realistic plan with a budget.

### The tele-psychotherapy call center

The tele-psychotherapy platform will be developed as an extension of The Medical Concierge Group (TMCG)'s existing tele-health services, also known as Rocket Health Africa. TMCG, a pioneering digital health entity, harnesses technology to offer a range of healthcare solutions including remote doctor consultations, community-based laboratory sample collection, doorstep pharmacy deliveries, and the creation of digital platforms for healthcare initiatives and research. For the prototype, the technical team will utilize detailed insights and schematic representations to craft the first version of the call platform. This will involve adapting the current infrastructure to introduce a mental health-specific toll-free line, ensuring community members can directly connect to the Rocket Health call center.

Upon dialing in, calls will be automatically redirected to available lay counselors, facilitating immediate start of the psychotherapy sessions. Following the initial setup, the developers will provide comprehensive orientation and training for the counselors involved in the project. These counselors, in turn, will educate their clients on navigating the call center access. Continuous enhancement of the call platform will be guided by user feedback, ensuring the service remains responsive to the needs of its clients.

### Standard mental health services (SMHS)

Standard mental health services provided at mental health treatment centers in Makerere University, Naguru, and Mulago, include a wide array of mental healthcare. However, mild to moderate mental health problems are addressed by mostly psychiatric clinical officers, nurses and counselors. Individuals seeking care will undergo physical and mental health assessments. Key services include unstructured counseling and or medication management for various mental disorders. For cases requiring specialized attention beyond the clinic's scope, referral systems ensure patients are directed to Butabika National Referral Mental Hospital.

### Sample size estimation

In a pilot trial the objective is not to prove superiority of the treatment but to test trial procedures and processes and to get estimates of parameters for the main trial sample size. According to Sim and Lewis et al, 2012, they propose a rule of the thumb for sample size of a pilot study which states that the sample size should be ≥55 and they do not put an upper limit (41). Other researchers argue that there is a trade-off, therefore, between having a small pilot study and a larger main trial or a larger pilot study and a smaller main trial. This is because the larger the pilot the more precisely estimated the variance will be and, hence, the smaller the inflation factor applied to the main study sample size calculation (42). For these reasons, we increased the sample size to 150 in each treatment arm.

### Study measures and data collection schedule

Study participants in the intervention and control arms will be asked to complete an interviewer-administered face-to-face or telephone-based standardized questionnaire to collect data on primary and secondary outcomes at baseline (T0), 6 months (T1), and 12 months (T2) after baseline.

### Sociodemographic variables

Sociodemographic variables will be assessed using a standardized socio-demographic questionnaire that will ask about descriptive information including age, gender, number of children, education, and relationship and employment status. Employment status will be categorized as “unemployed” and “employed”. Relationship status will be categorized as “never married”, “married/living with a partner”, and “divorced/separated”. Education status will be categorized as “secondary education”, “certificate”, and “diploma/degree”.

### Depression symptoms

Depression symptoms will be evaluated using the self-reporting questionnaire(SRQ-20) (43). Cross-cultural adaptation and validation of the SRQ-20 among PLWH in southern Uganda demonstrated that a cut-off score of six or higher provides optimal sensitivity (84%) and specificity (93%) for identifying current depression (44). This study will analyze SRQ scores as a continuous variable, and the scale's reliability will be assessed using Cronbach's alpha coefficient.

#### Suicide risk

The SAD PERSONS Scale (45) will be used in our study population because, despite its limitations and the lack of conclusive evidence for its predictive ability (46), it remains a widely recognized and practical tool for assessing suicide risk (47). Its simplicity and ease of use make it suitable for resource-limited settings where more sophisticated tools may not be readily available. Furthermore, incorporating it into the study will allow for the evaluation of its applicability and relevance in the African context, potentially contributing to the development of more tailored suicide risk assessment strategies in the future. Scores <4 will indicate low risk, 4–7 will indicate moderate risk, and ≥8 will indicate high risk, guiding intervention levels accordingly.

### Major depressive disorder

Major depressive disorder will be diagnosed using the MINI depression module, a structured diagnostic interview developed for the Diagnostic and Statistical Manual of Mental Disorders (4th Edition) (48).The psychometric properties of the MINI have not been formally evaluated in Uganda. However, the depression diagnostic section has been translated into Luganda, culturally adapted, and previously utilized in this context (49). The module will include two screening questions about depressed mood and loss of interest in daily activities over the past four weeks, seven additional questions about depression symptoms, and one question on functional impairment. A diagnosis will be made if the participant positively answers five or more symptom-related questions and the functional impairment question over a four-week period.

#### Anxiety symptoms

Anxiety symptoms will be assessed using the GAD-7 questionnaire (50). Although the GAD-7 has been validated in other African contexts (51, 52), it has not yet been validated in Uganda. This study will evaluate its reliability using Cronbach's alpha to determine its applicability in the study population. GAD-7 scores will be analyzed as a continuous variable within the study sample.

#### Alcohol use

Alcohol use will be assessed using the 3-item AUDIT-C, a shorter version of the AUDIT (53). The 3-item AUDIT-C, a shorter version of the Alcohol Use Disorders Identification Test(AUDIT), has not been validated in Uganda but evaluated and validated in several African contexts (54, 55). This tool will be used to assess risky alcohol use. Each question will be rated on a four-point scale, with total scores ranging from 0 to 12. A score of 4 or higher in men or 3 or higher in women will be recommended as an indicator of hazardous drinking behavior. The variable will be reported both categorically, using these cut-off points, and continuously, using total AUDIT-C scores.

#### Self-esteem

Self-esteem will be measured using the 10-item Rosenberg Self-Esteem Scale (56), with scores ranging from 10 to 40, where higher scores indicate higher self-esteem. The scale has not been validated in Uganda but has been evaluated and validated in several African contexts (57, 58). The scale's reliability will be assessed using Cronbach's alpha, and scores will be modelled as a continuous variable.

#### Disability days

Disability days will be assessed by asking: “How many working days have you lost due to depression-related symptoms in the previous 30 days?” Disability days reported will be modelled as a continuous variable. Single-item measures have shown good reliability and validity in assessing general health and physical activity (59).

#### Social support

Perceived social support will be assessed with the 12-item multidimensional social support scale (60). Perceived social support will be measured using the 12-item Multidimensional Scale of Perceived Social Support (MSPSS). This scale has been validated in Uganda, and its three-subscale structure—assessing support from family, friends, and significant others—has been confirmed (61). The scale uses a seven-point Likert format, with higher scores reflecting greater perceived support. Total scores will be calculated and analyzed as a continuous variable. The scale's reliability will be assessed using Cronbach's alpha for this study population.

#### Stigma

Stigma will be assessed using Nyblade and MacQuarrie's method, involving perceived, experienced, and internalized stigma (62) The tool is part of a broader effort to develop programmatic knowledge and interventions aimed at reducing stigma associated with various health conditions such as HIV, mental illness, and substance abuse. This tool is informed by a systematic review of literature and aims to fill existing gaps in stigma reduction strategies, particularly in health facilities. Scores will range from 0 (no stigma) to 40, with higher scores indicating higher levels of stigma. Scores will be modelled as a continuous variable.

#### Therapeutic alliance

The Scale to Assess the Therapeutic Relationships–Patient Version (STAR-P) will be used to evaluate the quality of therapeutic relationships from the patient's perspective, particularly in community mental health settings (63). It consists of 12 items divided into three subscales: positive collaboration, positive clinician input, and non-supportive clinician input. While not validated in Uganda, the STAR-P has been adapted into various languages for cultural relevance (64, 65) and has demonstrated strong psychometric properties, including good test-retest reliability (*r* = 0.76) and internal consistency, with high Cronbach's alpha values across diverse populations (66). The scale's reliability will be assessed using Cronbach's alpha for this study population.

#### Feasibility

Indicators of feasibility will be assessed by determining the proportion of eligible participants who engage in either intervention (reach), the proportion who attend all 8 sessions of either intervention (dose delivered), and the proportion lost to follow-up (attrition), using data from attendance registers.

#### Acceptability

The following indicators of acceptability including participant satisfaction, TSP counselor knowledge and attitudes, and the perceived effectiveness of the intervention in reducing depression will be evaluated using a 9-item questionnaire (67).

#### Fidelity

Fidelity will be assessed by asking supervisors to complete a semi-structured self-administered questionnaire to assess whether the interventions were delivered as planned.

Contextual influences will be assessed by asking TSP counselors to complete a semi-structured self-administered questionnaire to identify any facilitators or barriers to intervention delivery observed during group sessions. Additionally exit interviews with study participants will be conducted to assess participants’ knowledge, skills, or assets acquired during and after the interventions. This will provide insight into whether the interventions influenced targeted risk factors for depression.

### Determining cost-effectiveness

To assess the cost-effectiveness of providing TSP with SMHS vs. the provision of SMHS alone, we will adopt a societal perspective in our analysis. This approach encompasses not only the direct costs associated with the interventions—such as program delivery expenses—but also indirect costs like those stemming from the use of additional healthcare services and the loss of productive time due to participation in the interventions. We plan to devise a comprehensive questionnaire to gather detailed information on the utilization of supplementary healthcare resources, including visits to medical professionals covered by insurance, outpatient services, and expenditures on mental health medications, which may be influenced by the intervention.

The financial implications of implementing both the TSP & SMHS combination and the SMHS-only model will be meticulously calculated, focusing on contrasting these costs against the respective outcomes each model achieves, particularly regarding depression score improvements. It's crucial to clarify that the expense of conducting the research and any ancillary research activities, will be excluded from the cost-effectiveness evaluation.

Moreover, we intend to delve into the economic repercussions associated with time loss for different participant groups, including those volunteering without compensation, and the additional burden placed on individuals due to increased responsibilities linked to the study. To quantify these indirect costs, we will employ shadow pricing techniques. The identification of costs will be primarily based on project documentation and financial reports. For any costs not directly documented, we will turn to existing literature for estimates. Significant expenses related to services and procurements will be determined by the terms of reimbursement contracts or, in certain cases, prevailing market rates at the time of acquisition. Additionally, we will leverage databases such as the Uganda Bureau of Statistics (UBOS) to source pertinent data to inform our cost-effectiveness analysis, ensuring a robust and comprehensive evaluation of the interventions’ economic viability.

### Participant safety

During initial evaluations, participants will undergo thorough screening, and those for whom the interventions are considered medically unsuitable or hazardous will be excluded based on predefined criteria. Independent evaluators will monitor all participants for any adverse events at both 6- and 12-months post-intervention through a structured interview and report form. An adverse event (AE) encompasses any undesired or unintended sign, symptom, or medical occurrence linked to participation in tele-support psychotherapy, irrespective of its direct association with the therapy. Serious adverse events (SAEs) include any AE leading to outcomes such as death, a life-endangering situation, the necessity for hospital admission, or long-lasting or significant disability or incapacity. In this study, potential AEs might include suicide attempts resulting from an inadequate response to the tele-support psychotherapy sessions. A suicide management protocol is detailed in the study's risk management plan (see Supplementary File S2).

### Adverse event management

During the recruitment phase, potential participants exhibiting suicidal thoughts will undergo an assessment to evaluate their risk of suicide. Individuals diagnosed with depression who are identified as having a high risk of suicide will not be eligible for the study. Participants showing low to moderate suicide risk levels will be accepted into the study and will have their suicidal ideation evaluated at each counseling session. Should suicidal ideation persist after four counseling sessions, these participants will be directed to receive suitable care at the mental health departments of the hospitals involved in the study.

### Retention

To enhance adherence to the intervention sessions and maintain participant retention, lay health workers (LHWs) conducting the TSP sessions will receive a financial reward for their dedication to the project. Additionally, to re-engage participants who miss their TSP sessions, LHWs will be provided with airtime allowances to facilitate direct follow-up calls.

### Statistical analyses

The process evaluation of this pilot trial will adopt a mixed methods approach to thoroughly assess its primary outcomes, which include feasibility, acceptability, fidelity, the mediating processes behind observed effects, and the contextual factors acting as barriers or facilitators. For qualitative insights, accuracy checks will be performed on transcripts from Focus Group Discussions (FGDs) and Key Informant (KI) interviews, followed by translation into English and transcription. To ensure confidentiality, all interview data and notes will be anonymized and securely stored in the project's cloud-based folders. The qualitative data analysis will be conducted using MAXQDA Analytics Pro 2022 (Release 22.5.0), employing a grounded inductive approach to unearth recurring patterns and themes within the data. To enhance the analysis’ credibility, the thematic analysis will be collaboratively undertaken by at least three research team members. This collective effort aims to facilitate rich discussions, leading to a consensus on the emerging coding themes and a deeper understanding of the findings.

The interview data will initially be coded according to some themes corresponding to the focus questions. The codes will be used to construct matrix displays based on the co-occurrence of codes and the two treatment groups. The resulting matrix display will provide both the frequency and detailed content of responses, allowing us to assess how often responses varied between the two treatment groups. Inter-coder reliability will be assessed.

For the quantitative data, we will do bivariate analyses with *χ*^2^ tests and independent two-sample t tests to compare baseline demographic and psychosocial variables between study groups. Similarly, we will also do bivariate analyses to compare these variables between those who had completed all sessions (completers) and those who had not (non-completers).

We will assess randomization across the two arms by comparing socio-demographic characteristics and other potential confounding variables using chi-square for categorical variables and t-tests or other equivalent nonparametric tests, as appropriate, for continuous variables. Secondary outcomes will be the difference in follow-up mean depression, anxiety, stigma and social support scores between TSP-SMHS and SMHS (Control group) participants. These outcomes will be analyzed by ITT using generalized structural equation modelling. The STATA 16 statistical software will be used to conduct all analyses. Missing values will be imputed with multiple imputations.

### Cost-effectiveness analysis

The evaluation of cost-effectiveness will utilize depression scores as the primary metric. Depending on the accessibility of data, these scores will be connected to long-term indicators such as Quality-Adjusted Life Years (QALYs) or Disability-Adjusted Life Years (DALYs). This linkage will be facilitated through methodologies like WHO-CHOICE and the WHO's estimators for DALYs and QALYs (68). Adjustments will be applied to guarantee precision for factors like inflation, exchange rate fluctuations, and the discounting of both costs and outcomes.

The WHO-CHOICE (Choosing Interventions that are Cost-Effective) framework is designed to assess the cost-effectiveness of healthcare interventions by examining their effects on disease burden and related costs, providing a standardized yet adaptable approach for various health settings and conditions. Meanwhile, the WHO's DALY/QALY estimator measures the disease burden and the efficacy of health interventions in Disability-Adjusted Life Years (DALYs) or Quality-Adjusted Life Years (QALYs), taking into account both the years lost due to premature mortality and the years lived with disability or illness. These tools are invaluable for healthcare policymakers, aiding in the strategic allocation of resources and the development of cost-effective healthcare strategies.

To assess the cost-effectiveness of various service delivery models, our analysis will be aligned with established benchmarks, such as GDP per capita or findings from analogous studies in similar contexts. Throughout the study, we will comply with the economic evaluation principles outlined by the second panel on cost-effectiveness (69) and adhere to the CHEERS guidelines (70) for transparent and standardized reporting of our results.

### Ethical considerations

This study will seek approval from Makerere College of Health Sciences Research Ethics Committee and the Ugandan National Council of Science & Technology (UNCST). Researchers will gain full consent from hospital and community leadership prior to conducting the study. All participants will be required to give full informed consent to take part, and all identifying information fully anonymised in reporting. This trial is registered with The Pan African Clinical Trials Registry.

## Results

### Trial status

The Makerere School of Health Sciences Research Ethics Committee and the Uganda National Council of Science and Technology have granted approval for the trial. Recruitment commenced in May 2023 and concluded in July 2023, successfully enrolling 300 youths aged 15–30 years with mild to moderate depression levels. In parallel with the recruitment, TMCG developed the telepsychotherapy call platform. Those participants were randomly assigned to two groups: 154 were instructed to use their mobile phones to call the tele-psychotherapy platform to receive both Tele-support psychotherapy (TSP) and also to go to the nearest mental health treatment center for Standard Mental Health Services (SMHS) if their depression symptoms do not improve, and 146 were instructed to go to the nearest mental health treatment center and seek SMHS only. Currently, the analysis of data collected at the baseline is underway. The findings from this study are slated for presentation at both national and international forums. Efforts are also being made to draft manuscripts for submission to peer-reviewed academic journals, and comprehensive reports will be compiled for the funding body.

## Discussion

This study is expected to demonstrate the feasibility, acceptability, and preliminary effectiveness of Tele-Support Psychotherapy (TSP) delivered by lay counsellors via mobile phones to address depression among youth in Kampala, Uganda. By leveraging mobile health (mHealth) technologies, the intervention is anticipated to provide a scalable, accessible, and cost-effective mental health solution tailored to resource-limited settings. A key component of the study will be the development of a call platform, enabling youth with depression to connect with lay counsellors who will deliver eight sessions of therapy via mobile phones. Additionally, the integration of the Ecological Validity and Culturally Sensitive Framework is anticipated to enhance user engagement and ensure cultural relevance (71).

Other protocols have proposed single-session apps, gaming apps and virtual reality as digital mental health interventions for youth in sub-Saharan Africa. Osborn et al. explored the effectiveness of two digital single-session interventions (Shamiri-Digital and Digital-CBT) among Kenyan adolescents (11). While these single-session interventions show promise for scalability and accessibility, their practicality may be limited for addressing complex mental health issues like mild to moderate major depressive disorders, as effective problem-solving often requires emotional regulation—a process that takes time to develop (72).

In contrast, this protocol proposes a more practical and realistic approach by offering eight therapy sessions via mobile phones. The multi-session structure allows for a gradual and comprehensive therapeutic process: session 1 focuses on building rapport between the client and therapist, sessions 2 prepares the brain through psychoeducation, sessions 3 and 4 facilitate the release of strong emotions, and sessions 5 and 6 introduce positive coping strategies such as problem-solving. Sessions 7 and 8 further equip participants with income-generating skills to enhance resilience. This stepwise approach ensures that participants achieve emotional regulation before moving on to problem-solving, offering a more robust and sustainable therapeutic outcome.

Moreover, this protocol goes beyond the interventions of Shamiri-Digital and Digital-CBT by addressing barriers faced by underserved youth living in extreme poverty. Unlike smartphone-based apps, which may not reach those without advanced devices, this protocol includes the development of a call platform that will enable youth with basic phones to connect with lay counselors for therapy. Additionally, it will build mental health capacity by training lay counselors to deliver the intervention, creating a sustainable support system within the community. By addressing contextual and infrastructural limitations, this protocol offers a more comprehensive and inclusive solution tailored to the realities of resource-limited settings.

Our proposed study offers key advantages over other studies using gamified mental health apps (12). Unlike the apps, which rely on smartphone access and provide brief, self-guided behavioural activation, TSP will use a call platform accessible to youth with basic phones, ensuring broader reach among underserved populations. TSP sessions will be similar to GSP sessions with the goal of delivering a comprehensive therapeutic process, addressing emotional regulation, psychoeducation, coping skills, and resilience-building, which is more practical for managing complex mental health issues. Additionally, TSP will incorporate human interaction through trained lay counselors, fostering trust and adaptability, and will employ a rigorous mixed-methods evaluation to assess both feasibility and acceptability and effectiveness and contextual mechanisms. This inclusive, scalable approach positions our TSP intervention as a potentially more impactful and sustainable solution for youth mental health in resource-limited settings.

Similarly, our protocol offers advantages over virtual reality (VR). For example, the Mobile Health–Supported Virtual Reality and Group Problem Management Plus (PM+) protocol by Logie et al. (4) also targets urban youth but relies on VR and group-based PM + therapy (73). While VR may be less accessible to youth living in extreme poverty, our TSP study will use a call platform compatible with basic phones, ensuring it can reach a broader audience. TSP prioritizes scalability and cost-effectiveness by leveraging simple mobile technology and training lay counsellors, making it better suited for addressing depression among diverse youth populations in low-resource settings.

This study's findings can potentially transform mental health care delivery in low- and middle-income countries (LMICs). By demonstrating the viability of tele-psychotherapy, this research could inform policies and guide the integration of digital mental health interventions into primary care systems. The anticipated success of the TSP model could help bridge gaps in mental health care access, reduce stigma, and address provider shortages in resource-constrained settings. Furthermore, the study could lay the groundwork for scalable, cost-effective, adaptable digital interventions across diverse cultural and socio-economic contexts.

Several limitations must be acknowledged. As a pilot randomized controlled trial (RCT), the findings may have limited generalizability to broader populations. Challenges such as inconsistent network access, varying familiarity with digital tools among participants, and cultural factors influencing acceptability may affect recruitment, retention, and adherence to the intervention. Additionally, the reliance on self-reported measures for outcomes and use of measures with low predictive values such as the SAD PERSONS scale (74) may introduce bias.

## Conclusion

This protocol represents a significant step forward in addressing youth mental health challenges in resource-limited settings. Combining innovative technology with evidence-based psychotherapy offers a practical and scalable solution to bridge gaps in mental health care delivery. While limitations exist, the study's anticipated findings have the potential to inform future research, policy, and practice, ultimately advancing mental health care equity for underserved populations. Through a balanced approach acknowledging opportunities and challenges, this protocol sets the stage for broader adoption and adaptation of tele-psychotherapy interventions in low-resource settings.

Future protocols should expand on this study by conducting larger, multi-site trials to validate the scalability and generalizability of the TSP model. Research should also explore integrating advanced features, such as artificial intelligence-driven customization and real-time monitoring, to enhance engagement and outcomes. Long-term studies are needed to evaluate the sustained impact of tele-psychotherapy on mental health outcomes and its economic viability in diverse settings. Moreover, incorporating feedback loops with participants and community stakeholders will further refine and adapt the intervention.
