# Supplementary material for: Developing and testing tele-support psychotherapy using mobile phones for depression among youth in Kampala district, Uganda: study protocol for a pilot randomized controlled trial
Source: Front Digit Health. 2025 Feb 18;7:1515193. doi: 10.3389/fdgth.2025.1515193 (PMC11876404; doi:10.3389/fdgth.2025.1515193)
Supplement: Supplementary file 1 [file Datasheet1.pdf]

Risk management plan

Below, we describe the risks to project success and our methods to reduce these risks.

1. The proposed research will be conducted according to guidelines provided by the Uganda National Council of Science and technology (UNCST). The guidelines require all researchers to conduct research activities while observing Covid-19 prevention guidelines provided by the Ministry of Health (MOH). Further, all research projects are required to submit risk management plans alongside their project proposals to the research ethical committee for review. Our risk management activities will include

   Working with only fully vaccinated project staff; conducting daily temperature screens for all research team members and study participants, training research teams in Covid-19 MOH prevention guidelines; providing educational materials on Covid-19 prevention to all study participants recruited into the project; ensure availability of personal protective equipment such as face masks, water and soap for hand washing at all research offices and field sites. Research assistants will ensure at least 2m physical distance between them and study participants during screening and interview procedures. These plans will ensure timely identification, isolation and referral of suspect cases to the District Covid-19 Task Force Team. We have budgeted for PPE and other items needed for infection prevention and control such as hand sanitizers and surface disinfectants.

2. **Prevailing stigma towards mental health problems and fear of utilizing mental health services.** Preliminary research and experience in the mental health sector in Uganda has shown that mental health stigma is widely entrenched especially in health workers. As a result, fear of exclusion prevents people from sharing common mental health problems, or from seeking treatment. Our team believes that this can be combatted through mental health sensitization, educating the community and informing people that it is possible to recover from mental health problems. It will also be emphasized that trained health workers and health professionals can

help people manage common mental health problems by offering counseling as a first line treatment and where there is no improvement, referrals for consultation and medication will be made.

3. **Accommodating varying explanatory models of mental illness**. Based on preliminary research on mental health awareness in Uganda, many participants attribute mental illness to social and supernatural causes such as witchcraft. They would not typically seek advice from health workers about mental health problems such as depression, or anxiety. Many prefer to handle common mental health problems personally through prayer, seeking advice within their families, communities, local or religious leaders. Tele-support psychotherapy will be presented with consideration of this context as a new alternative, which will work alongside and in support of existing medical and non-medical care models.
4. **Health Worker Retention.** People recruited from the relevant communities to act as lay counsellors may have various competing responsibilities and therefore retention can be a challenge. This will be mitigated through an intensive selection process based on recommendations from the district health officer and health center managers. An adequate reimbursement strategy will also be fully considered in order to incentivize on-going participation.
5. **Breach of confidentiality:** The primary risk to the participants is a breach of confidentiality. The following steps will be taken to prevent this. Personnel are expected to follow procedures to protect confidentiality of information obtained during counselling sessions, and that contained on the individual study forms. The data will be collected and managed using tablet-based electronic forms; forms will be created and data will be managed using RedCAP. Databases will be kept on a secure, encrypted server and data systems will be designed to exclude transmission of personal identifiers to persons uninvolved in the study. Records will be identified by participants' study ID numbers. Once received, identifiable data will not be released to anyone outside those involved the study. Training of health workers will emphasize the maintenance of confidentiality in all counselling sessions except for situations where a study participant reveals intent to harm him/her or others. To mitigate such extreme events extra caution to adhere to the inclusion /exclusion criteria. Only study participants with mild to moderate common mental health problems

with low to moderate suicide risk will be recruited into the study. Furthermore, we will provide all individuals with contact information for medical personnel and the study team, and ensure that all participants are aware of the hospital resources available to them.

6. **Experience of Distress:** Some risks inherent in any mental health care may result in response to the assessment measures, since instruments will ask about thoughts, feelings, and personal difficulties that may be private. Risks also include possible embarrassment, distress, or inconvenience related to questions regarding personal information and mental health problems. We will take multiple measures to minimize this distress of clients. First, assessors will be trained in responding to distress. Therefore, trained health workers will be available to recognize such distress and refer to the mental health workers. In the event that any individual is experiencing significant distress, the trained health workers will notify the mental health workers at Kitgum General Hospital and will ensure that the affected individual receives appropriate counselling. Training of health workers will take place before recruitment of study participants. Clients will also be told that they can refuse to answer any questions and still receive services.
7. **Suicidal Intent:** Study participants who reveal suicidal intent during the course of the study. Although this is not a direct risk with participation of this study, and thus a low likelihood, it is important to have a plan in place, particularly in a low-resource setting. All lay counsellors will be trained specifically in how to assess for suicide risk, and the procedure to follow if there is intent. The procedure is based on a locally-developed safety protocol outlined below.

Suicide safety protocol

Lay health workers will be trained to follow these steps when a client expresses a wish to end their life.

a) Validate the client's suicide feelings by saying: *Indeed, the problems you have shared with us are very hurting and painful. It is not surprising that they have caused you to have thoughts about taking your own life. OR (In case client expresses suicide intentions at the start of counseling session);Say: It seems whatever you are going through must be very hurting and painful*

b) Find out if the client has a definite plan: *Had you thought about the date and specific means to do this? Had you bought the rope? Poison? Have you written a goodbye note?*

c) In case client has not made any plans, you say; *It is good you have not made any plans.*

d) When the client has made and revealed plans, respond; *Even though you have made those plans, remember that we are meeting so that you can get guidance on how to solve our problems.*

e) Assess the suicide risk of the client **(SADPERSONS)**

- **S**- Male = 1female = 0
- **A** – Age (if the client is a young male (15-25 years) or elderly, = 1
- **D** - Depressive thoughts = 1
- **P**- Previous attempt yes = 1
- **E** – Ethanol (use of alcohol) =1
- **R** – Rational (ever lost touch with reality; e.g. hearing voices others don't hear) yes = 1
- **S** – Social support (are there people he/she can turn to for support) no= 1
- **O**- Occupation (unemployed) = 1
- **N**- No spouse = 1
- **S**- Chronic sickness = 1

Less than ≤ 4 = low (mild) risk

5-7 is moderate risk

8-10 is severe risk

f) If the risk is mild to moderate, you proceed with the tele- support psychotherapy.

g) If the risk is severe, the trained health worker will alert mental health workers at The Kitgum general hospital to take up management of the affected young person and their caregiver..

h) When the trained health worker decides to continue with the psychotherapy for those with mild and moderate risk, they will remind the young person of the commitment to attend all 8 sessions. *Say: If you take your life, you will be breaking that commitment. If you take your life, we will not be able to give you guidance. We need you alive so that we can help you.*

i) We need you alive so that we may complete this journey (8 sessions of tele-support psychotherapy) together as we committed earlier. Can you consider completing this journey with me?

j) Group members will be asked to check on the affected young person during the week and the trained health worker assess for suicidal thoughts in every session to establish if the thoughts are persistent or remitting.

Other Adverse Events: Lay counsellors will be trained to screen for adverse events. When an adverse event occurs, an Adverse Events Report Form will be filled out by research assistants. Once the participant has been properly tended to, the adverse events will be reported immediately to the on hand field coordinator and to the study administrators. The IRB will be notified of each serious adverse event as it occurs. Reportable adverse event forms will be submitted to the research ethics committee at Makerere University. Serious adverse events will be reported by telephone or e-mail within 24 hours of discovery of the event

Dissemination of Results: The study's findings will be communicated to a wide audience, including mental health service providers and policymakers in Uganda, such as the Ministry of Health and the Makerere School of Medicine Department of Psychiatry. International bodies like USAID, the Mental Health Innovation Network, the Wellcome Institute, and the UCL Institute for Global Health will also be informed. Research outcomes will be published in open-access journals and presented at relevant academic conferences. Participants will receive updates in their preferred format, such as workshops or written summaries. Additionally, the results will be used to advocate for the adoption of cost-effective digital mental health solutions and address socio-economic factors affecting mental health.
